# Biomechanical testing of a unique built-in expandable anterior spinal internal fixation system

**DOI:** 10.1186/1471-2474-15-424

**Published:** 2014-12-11

**Authors:** Chu-Song Zhou, Yan-Fang Xu, Yu Zhang, Zhong Chen, Hai Lv

**Affiliations:** Department of Orthopaedics, Zhujiang Hospital of Southern Medical University (First Military Medical University), Guangzhou, 510282 People’s Republic of China; Department of Orthopaedics, The Second People’s Hospital of Changzhi City, No 83 Speace West Street, Changzhi City, Shanxi 046000 People’s Republic of China; Department of Orthopaedics, Guangzhou General Hospital of Guangzhou Military Command, Guangzhou, 510010 China

**Keywords:** Thoracolumbar, Anterior fixation, Expansion screw, Internal fixation, Biomechanical, Pullout strength

## Abstract

**Background:**

Expandable screws have greater pullout strength than conventional screws. The purpose of this study was to compare the biomechanical stability provided by a new built-in expandable anterior spinal fixation system with that of 2 commonly used anterior fixation systems, the Z-Plate and the Kaneda, in a porcine partial vertebral corpectomy model.

**Methods:**

Eighteen porcine thoracolumbar spine specimens were randomly divided into 3 groups of 6 each. A vertebral wedge osteotomy was performed by removing the anterior 2/3 of the L1 vertebral body and the T15/L1 disc. Vertebrae were fixed with the Z-Plate, Kaneda, or expandable fixation system. The 3-dimensional spinal range of motion (ROM) of specimens in the intact state (prior to osteotomy), injured state (after osteotomy), and after internal fixation were recorded. The pullout strength and maximum torque of common anterior screws, the expandable anterior fixation screw unexpanded, and the expandable anterior fixation screw expanded was tested.

**Results:**

After internal fixation, the expandable device and Z-plate system exhibited higher left bending motion than the Kaneda system (5.50° and 5.37° vs. 5.04, p = 0.001 and 0.008, respectively), and the Z-plate and Kaneda groups had significantly higher left axial and right axial rotation ROM as compared to the expandable device group (left axial rotation: 5.23° and 5.02° vs. 4.53°; right axial rotation: 5.23° and 5.08° vs. 4.49°). The maximum insertion torque of the expandable device was significantly greater than of a common screw (5.10 vs. 3.75 Ns). The maximum pullout force of the expandable device expanded was significantly higher than that of the common screw and the expandable device unexpanded (3,035.48 N vs. 1,827.38 N and 2,333.49 N).

**Conclusions:**

The built-in anterior fixation system provides better axial rotational stability as compared to the other 2 systems, and greater maximum torque and pullout strength than a common fixation screw.

**Electronic supplementary material:**

The online version of this article (doi:10.1186/1471-2474-15-424) contains supplementary material, which is available to authorized users.

## Background

Surgical procedures for the treatment of thoracolumbar spine diseases include anterior, posterior, and combined anterior and posterior fusion, and adequate immobilization of the spine is critical for successful fusion. The use of anterior thoracolumbar implants and anterior instrumentation for stabilization has become more common in the past decade for reasons including direct visualization and ready access to the anterior column, improved distraction, and placement of a large interbody fusion device [1, 2]. At present, there are 3 systems used for anterior thoracolumbar fixation [1]. One is the screw-rod system, which is represented by the Kaneda® SR Spine System (DePuy Synthes), another is the screw-plate system, which is represented by the Z-Plate Atl™ Anterior Spinal Fixation System (Sofamor Danek) system, and the last is a screw system, which is represented by the MACS HMA (Aesculap, Tuttlingen) system and consist of porous hollow titanium screws with an outer diameter of 12 mm for monocortical use [3]. Though all systems provide adequate anterior fixation, they all have unique strengths and weaknesses.

While the screw-rod system is relatively stable biomechanically, the size is relatively large and the implant profile is relatively high, which can damage peripheral neurovascular structures during or after surgery [1, 4]. In addition, the screw-rod can only be used for vertebrae superior to L3 due to the psoas muscle and pelvic ring. The screw-plate system is relatively simple in structure, easy to manipulate during surgery, and is associated with a relatively short operative time. However, its biomechanical stability is poorer than the screw-rod system, and the plate is less likely to completely match the lateral side of the vertebral body and often leaves gaps [1]. In addition, stress is concentrated at the locking site between the plate and the screw, which can result in internal fixation failure and kyphosis during the late stage after surgery [1]. Although the screw system overcomes the shortcomings of the screw-rod system and the screw-plate system to some extent, the screw has a large diameter (12 mm), and it cannot be used for people with small vertebrae, especially children and adolescents [3].

Expandable screws have been used for improved graft fixation in anterior cruciate ligament reconstruction [5]. Richter et al. [6] reported that a monocortical expansion screw provided the same biomechanical stability as a bicortical 3.5-mm AO screw and better biomechanical stability than a cervical spine locking plate when used in anterior internal fixation of the cervical spine. Studies have also shown that expandable pedicle screws provide improved pullout strength as compared to standard pedicle screws, especially in osteoporotic bone [7–9].

We designed a built-in expandable anterior thoracolumbar fixation system to overcome the shortcomings of current anterior thoracolumbar systems. The fixation system is made of a titanium alloy, and composed of an expandable cylinder screw with an internal cone-shaped space. When an insert is placed into the internal space of the screw, the distal end of the screw expands. The purpose of this study was to compare the biomechanical stability provided by the new anterior fixation system with that of 2 commonly used anterior fixation systems, the Z-Plate and the Kaneda, in a porcine partial vertebral corpectomy model.

## Methods

### Evaluation of 3-dimensional (3D) spinal range of motion (ROM)

**Figure 1:**
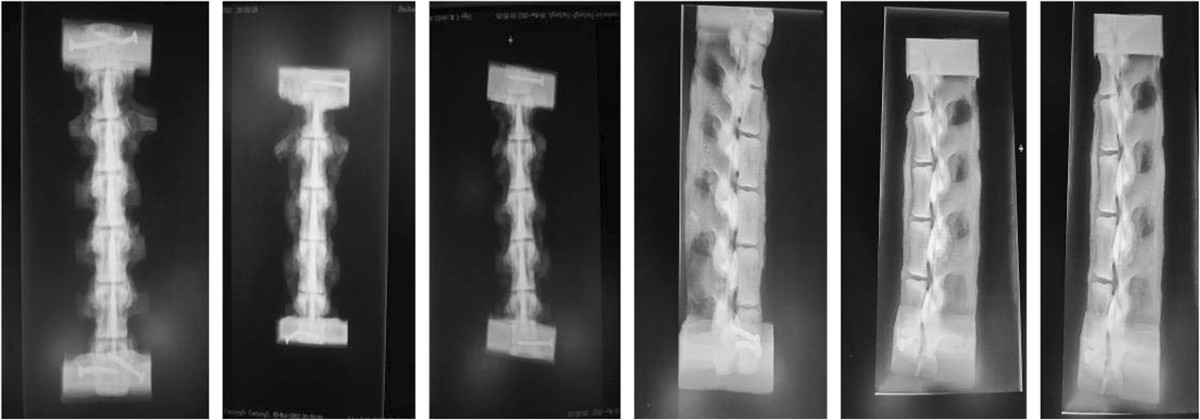
**Radiographs of thoracolumbar spine specimens used for the experiment.**

A total of 42 fresh pig thoracolumbar spine (T14-L3) specimens were obtained from a local slaughterhouse, all animal experiments were carried out with the approval of the Southern Medical University Animal Care and Use Committee in accordance with guidelines for the ethical treatment of animals and 18 out of the 42 specimens similar in size were selected for testing ROM. Specimens with an intact posterior ligament complex (PLC) were choosen. Congenital diseases, osteoporosis, tumors, and fractures were excluded by visual observation and anteroposterior and lateral radiographies (Figure 1). All soft tissue was removed, and the T14 and L3 vertebrae were embedded in polymethyl methacrylate. During embedding, the L1 vertebra was placed in a standard location for axial and torsional loading. The experimental specimens were prepared to make parallelism errors of the upper- and lower-end planes of the denture powder platform ≤ 1°, sealed with double-layer plastic wrap, and preserved at -20°C until use [10]. The specimens were removed from the freezer 10 hours before experiments, and thawed at room temperature [11].

A vertebral wedge osteotomy was performed by removing the anterior 2/3 of the L1 vertebral body and the T15/L1 disc with a steel saw as described by Panjabi et al. [12] to produce a model with an anterior and middle column compression injury in the L1 vertebra. The bone fragments were preserved for later bone grafting with a titanium mesh. Specimens were considered satisfactory for use as a fracture model if 50% of the vertebral height remained, and lower endplate of T15 and the upper endplate of L2 were intact. Specimens were excluded if fractures of T15 or L2 were present, there was injury to the posterior column of the L1 vertebra, or there were fractures of the lower endplate of T15 or upper endplate of L2.

**Figure 2:**
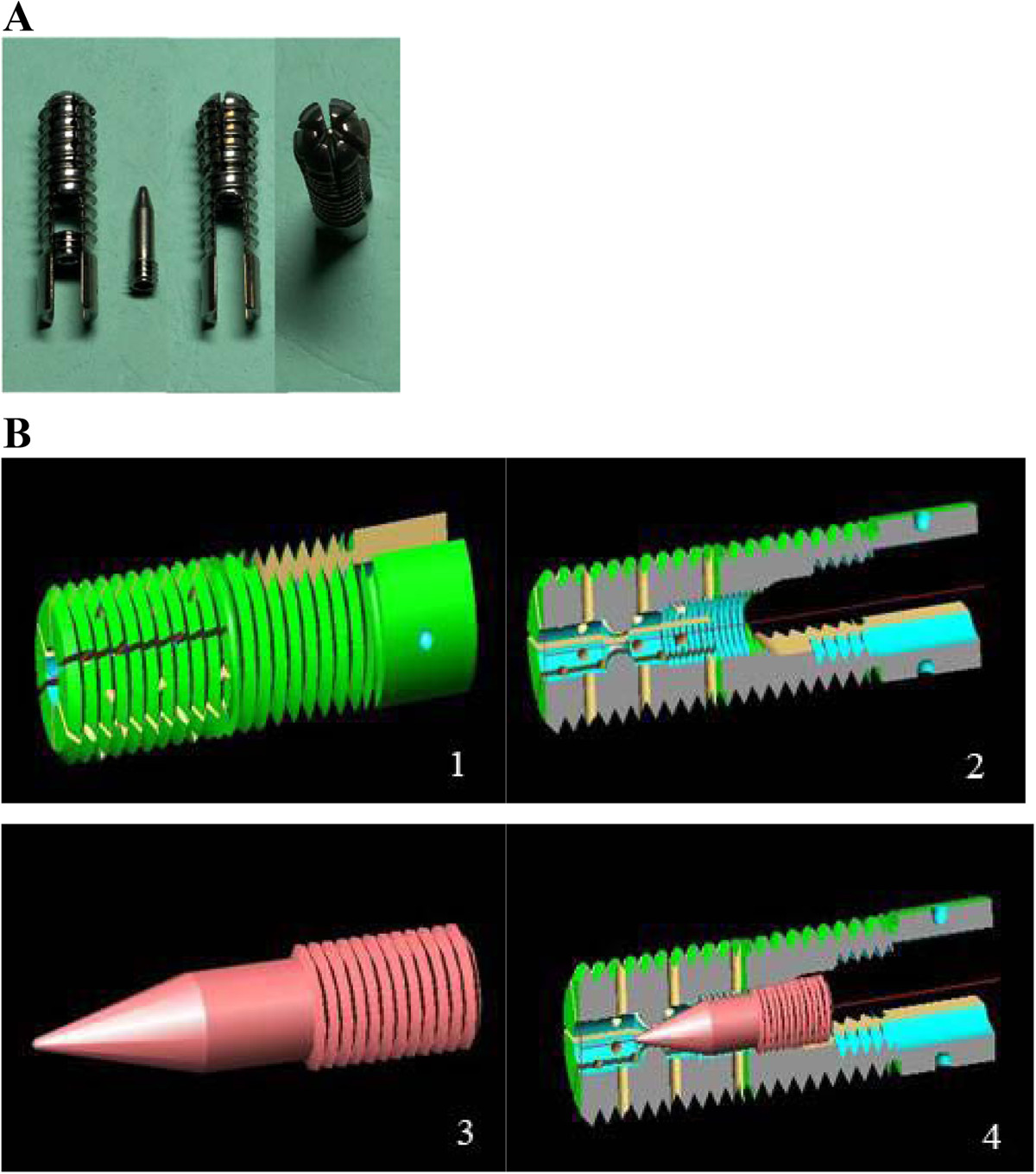
**Actual picture and diagrammatic illustrations of the fixation system. A)** The built-in expandable anterior spinal fixation system. **B)** Diagrammatic illustrations of the fixation system. 1) Exterior view 2) lateral interior view (without insert); 3) insert; 4) with insert placed to expand the screw. After the insert is placed to expand the screw, the diameter of the expanded tip is 9.0 mm inside the vertebral body.

**Figure 3:**
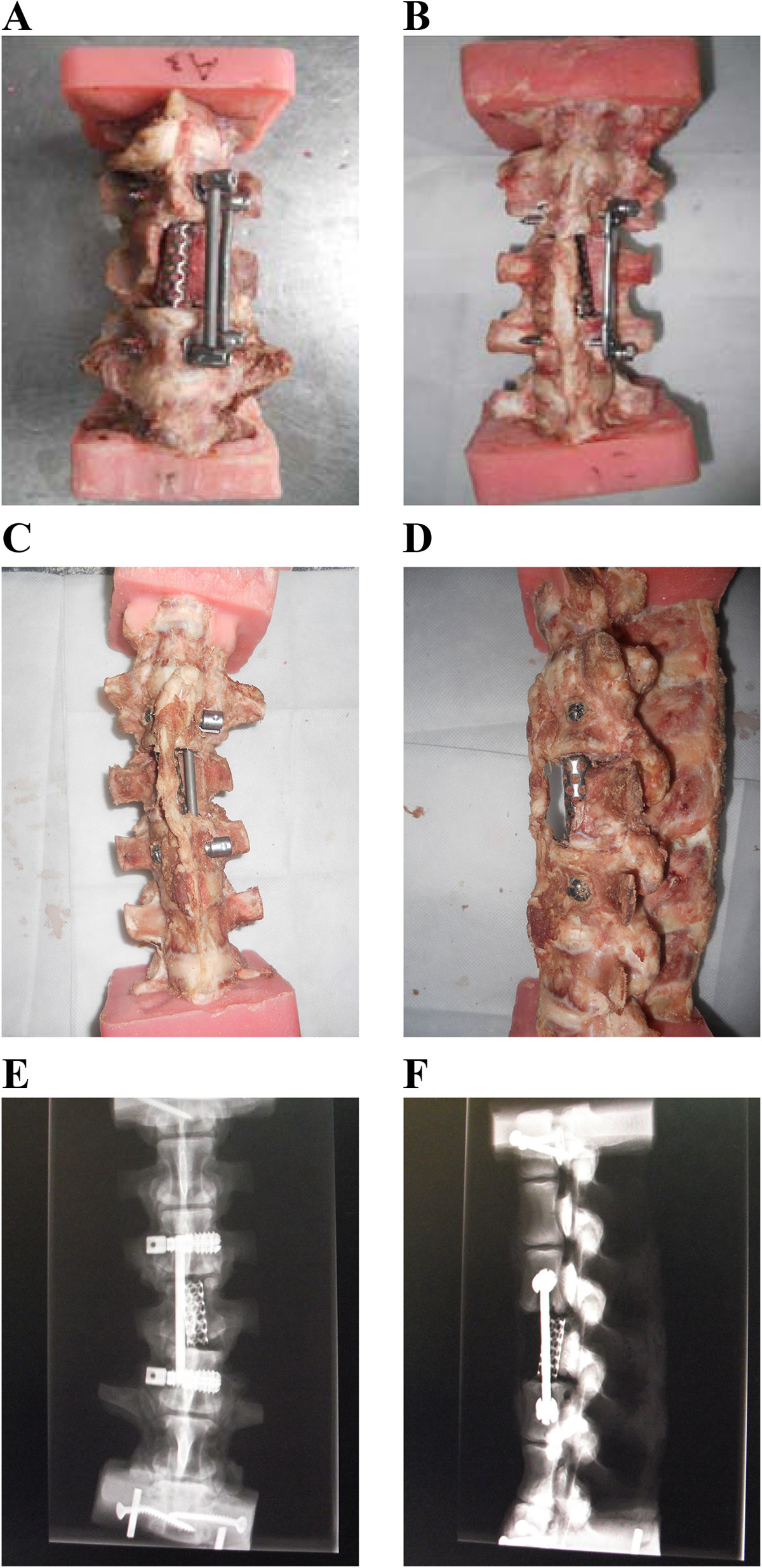
**Pig thoracolumbar specimens fixed with the Kaneda system (A), the Z-Platesystem (B), and the built-in expandable anterior spinal fixation system (C-F).** Images **E** and **F** are radiographs of images **C** and **D**, respectively.

The 18 specimens were randomly divided into 3 groups with 6 specimens in each group. A different fixation system was used in each group of 6 specimens: 1) Built-in expandable anterior spinal fixation system (Patent No. ZL2012200592496; Beijing Fule Technological Development Co., Ltd) (Figure 2); 2) Screw-rod (Kaneda) system; 3) Screw-plate (Z-Plate) system. When performing posterior internal fixation the posterior stabilization system of the spine and peripheral tissues and ligaments were preserved as much as possible to maintain spine stability. Nine K-wires 2.0 mm in diameter were inserted into the spinous processes and bilateral transverse processes of the T15, L1, and L2 vertebrae of the embedded specimens (3 wires in each vertebra). Each K-wire was fixed with a spherical marker for tracking its 3D motion. During the experiment, the specimens were sprayed with normal saline to maintain the viscoelasticity of the tissue [13]. Images of the specimens fixed with the 3 different systems are shown in Figure 3.

**Figure 4:**
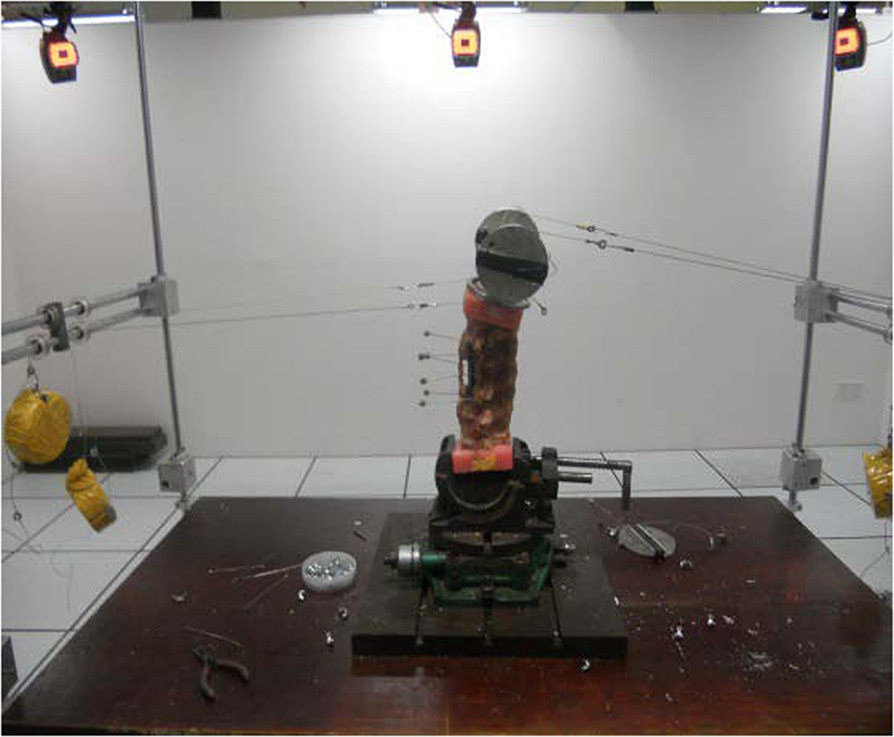
**Device used to measure 3-dimensional spine motion.**

For testing, the upper end of the embedded specimen was connected to the loading tray of a 3D motion testing device (Southern Medical University), and the lower end was fixed to the base of the testing device. Testing was carried out according to the method described by Wilke et al. [13]. Flexion, extension, left lateral bending, right lateral bending, left axial rotation, and right axial rotation were carried out for each group. Pure moment loading was used for all specimens; the load was 6.0 N^.^m [14], and 30 s was taken as 1 cycle. Before measurements each motion, the maximum torque (6.0 N^.^m) was loaded and then unloaded in each specimen, and the process was repeated 3 times. Data were collected after 3 cycles of loading/unloading to minimize the influence of viscoelasticity of the spine, and obtain relatively stable results of thoracolumbar motion. The loading was maintained for 30 s at 6.0 N^.^m, and creep motion of the specimen was allowed. The loading test was carried out at room temperature (20-30°C). An image of the device used to test spinal motion is shown in Figure 4.

The 3D ROM of specimens in the intact state (prior to osteotomy), injured state (after osteotomy), and after internal fixation were recorded. Briefly, 6 infrared cameras (Hawk digital cameras) were placed around the specimen. An Eagle-4 digital infrared camera was used to dynamically trace and capture the displacement of the spherical makers fixed on the specimen (accuracy = 1 nanometer, time interval = 0.001 s). Images of each moving vertebra with spherical makers on the same plane, which can accurately reflect the range of vertebral motion, were selected. The displacement of each segment was calculated using EVaRT software and based on the principle of optical 3D motion analysis.

Image analysis and data conversion were carried out using the Geomagic software system. Angular displacement of each vertebra was calculated according to the coordinates of the 3 non-collinear markers on each vertebra. The range of motion was calculated by adding the angular displacement of each vertebra. The parameters of the ROM of adjacent thoracolumbar segments were: Neutral zone (NZ), the ROM between the spine location at zero load and the neutral location; Elastic zone (EZ), the ROM between the neutral location and the location at maximum load; Spine ROM, the ROM between the spine location at zero load and that at maximum load (the sum of NZ and EZ) [15]. Because it is difficult to identify the ROM of the neutral location, we assumed that this location was the center between the locations at positive and negative force couples.

### Pullout strength and maximum torque

Twenty-four of the 42 spine specimens were divided into 4 groups (group A-D, n = 6 each group) for testing pullout strength and maximum torque. In group A, the pullout strength and maximum torque of common anterior screws (diameter, 5.5 mm; thread length, 30 mm) was tested. In group B, the pullout force and maximum torque of the expandable anterior fixation screw (diameter, 12 mm; thread length, 30 mm) unexpanded was tested. In group C, the maximum torque of the expandable anterior fixation screw expanded was tested. In group D, the pullout force of the expandable anterior screw expanded was tested. Testing was performed in the Biomechanics Laboratory of Southern Medical University using a Bose ElectroForce® BioDynamic® test instrument, which automatically records the load signals.

**Figure 5:**
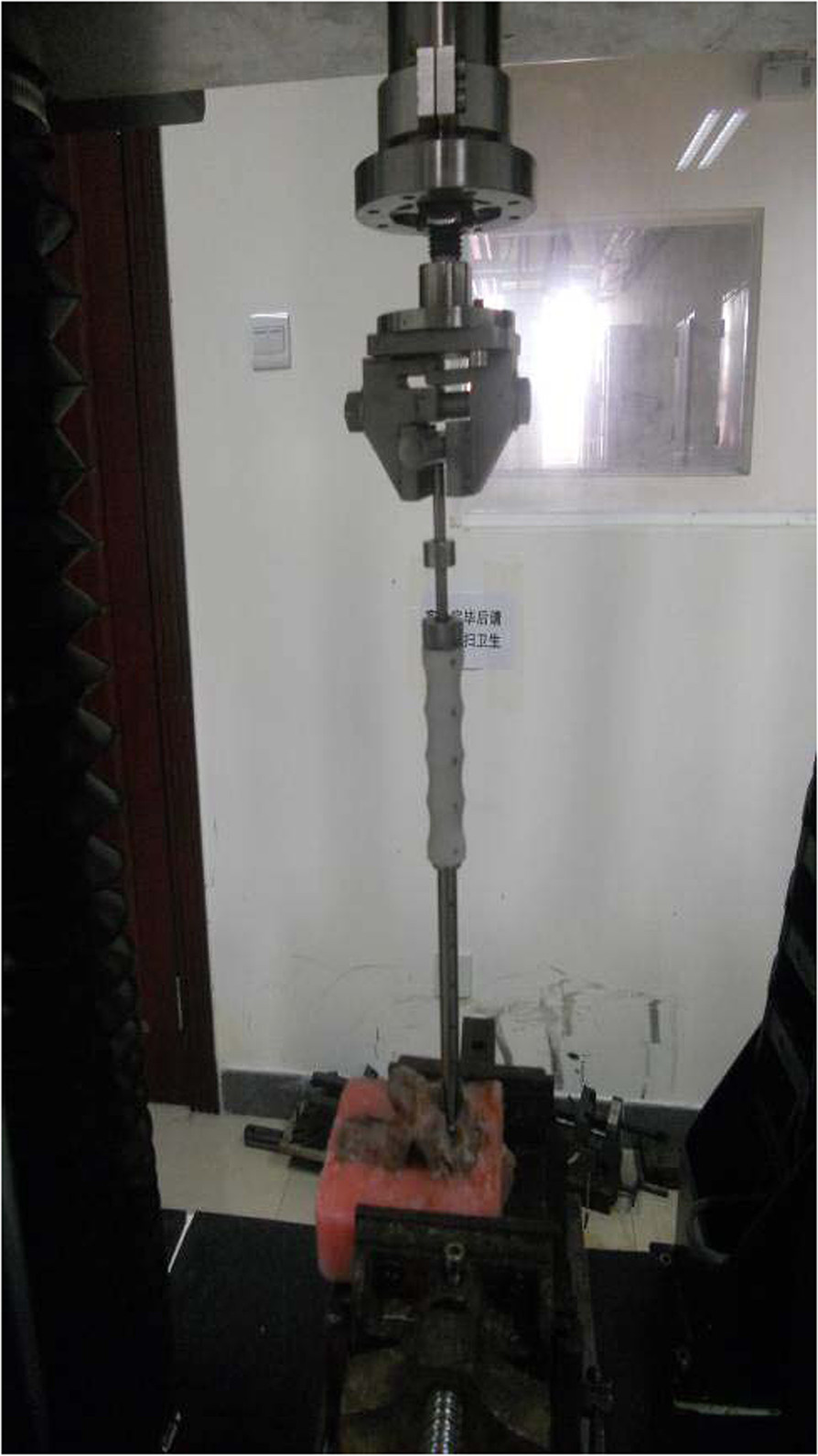
**Device used for maximum insertion torque test.**

**Figure 6:**
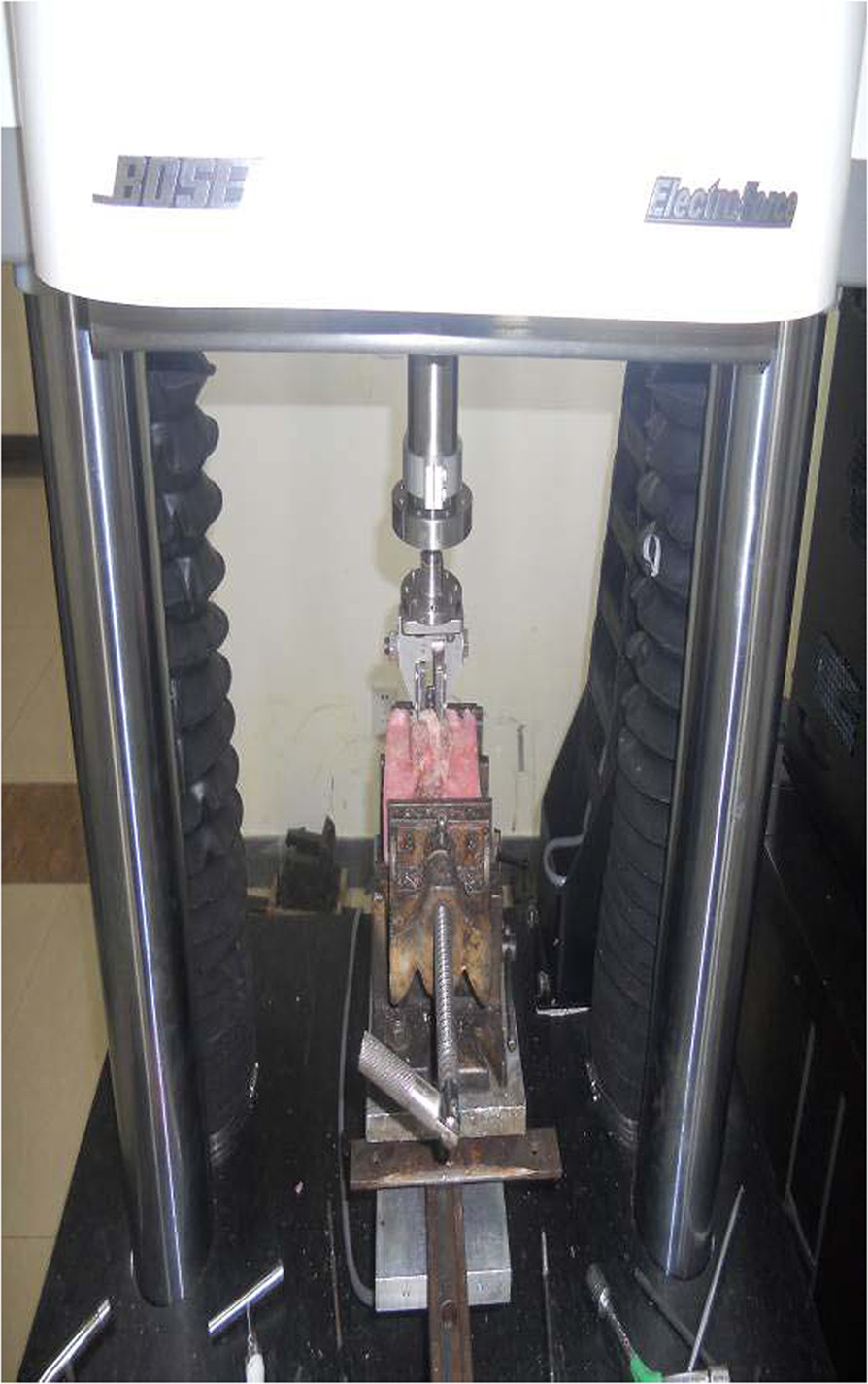
**Device used for maximum pullout strength test.**

Screws were fixed according to their standard installation methods. In group A and B, the screw was fastened to the last half of the thread. In the group C and D, the screw was fastened to the whole thread, bone fragments were placed inside the screw, and the inner core was placed and the screw was expanded. The specimen was then embedded again. In cases in which the screw penetrated the contralateral cortex, the protruding portion of the screw was embedded with plasticine first and then embedded and fixed with denture powder to prevent the denture powder from fixing the screw.For torque testing, specimens were fixed to special fixtures after internal fixation, and the screw adjuster was fixed to the testing device (Figure 5). The angle of the specimen was adjusted to make the testing device, screw adjuster, and the direction of screw insertion lie on a straight line. A 90° clockwise rotation (groups A and B) or 90° counter-clockwise rotation (group C) of the specimen was carried out at 240°/min angular velocity, and the load at 90° rotation was recorded. The peak value was defined as the maximum insertion torque of the screw. For testing pullout force, the specimens were fixed and the angle adjusted as described above (Figure 6). The pullout test was carried out at a loading rate of 1 mm/min, and stopped if the screw broke. The standard of confirming screw breakage is the appearance of the highest point on the load-deformation curve. The peak value was recorded as the pullout force of the screw.

### Statistical analysis

All testing was performed, and results confirmed by agreement of the 2 primary authors, each with more than 15 years of experience. ROM, maximum insertion torque, and maximum pullout strength were presented by mean and standard deviation. One-way ANOVA was performed to compare continuous data among independent groups. The least-significant difference (LSD) method was used for further multiple comparisons if one-way ANOVA provided statistically significant results. Paired t-test was performed to compare the ROM between normal vs. injured, normal vs. internal fixation, and injured vs. internal fixation status within groups. Data were examined with the Kolmogorov-Smirnov test for normality. Statistical analyses were performed using SPSS 15.0 statistical software (SPSS Inc., Chicago, IL). A 2-tailed p < .05 indicated statistical significance.

## Results

### Spinal ROM

The Kolmogorov-Smirnov test for normality indicated that all data were normally distributed (Additional file 1: Table S1). Similar changes in the ROM between the normal, injured, and internal fixation states in the 3 groups was noted (all, p >0.05) (Table 1). The ROM after injury was significantly increased in each group as compared to the normal state (all, p <0.001). After internal fixation, the ROM in all groups was significantly decreased as compared to the injured state (all, p <0.001).

**Table 1:** **Spinal range of motion of the 3 fixation systems**

ROM (°)	Status	Expandable device (n = 6)	Z-plate (n = 6)	Kaneda (n = 6)	p
Flexion	Normal	9.64 (0.81)	9.98 (1.34)	9.49 (0.94)	0.717
	Injury	17.73 (3.67)^a^	19.02 (3.12)^a^	19.18 (3.21)^a^	0.718
	Internal fixation	5.72 (0.44)^ab^	5.70 (0.50)^ab^	5.63 (0.65)^ab^	0.957
Extension	Normal	8.45 (0.52)	8.45 (0.43)	8.48 (0.44)	0.994
	Injury	13.51 (2.21)^a^	13.50 (2.53)^a^	13.43 (2.25)^a^	0.998
	Internal fixation	5.25 (0.28)^ab^	5.14 (0.31)^ab^	5.12 (0.19)^ab^	0.671
Left bending	Normal	8.71 (0.30)	8.78 (0.27)	8.55 (0.41)	0.484
	Injury	18.07 (3.66)^a^	18.23 (3.81)^a^	18.87 (4.22)^a^	0.932
	Internal fixation	5.50 (0.21)^ab^	5.37 (0.21)^ab^	5.04 (0.13)*†^ab^	0.002
Right bending	Normal	8.35 (0.37)	8.47 (0.42)	8.43 (0.39)	0.869
	Injury	18.45 (3.72)^a^	18.69 (3.80)^a^	19.05 (2.54)^a^	0.955
	Internal fixation	5.01 (0.12)^ab^	5.05 (0.14)^ab^	5.04 (0.11)^ab^	0.887
Left axial rotation	Normal	7.11 (0.40)	6.97 (0.34)	7.07 (0.28)	0.769
	Injury	19.56 (2.98)^a^	19.05 (3.22)^a^	19.04 (3.26)^a^	0.948
	Internal fixation	4.53 (0.42)^ab^	5.23 (0.30)*^ab^	5.02 (0.15)*^ab^	0.005
Right axial rotation	Normal	6.95 (0.35)	6.91 (0.31)	6.84 (0.44)	0.883
	Injury	19.60 (3.18)^a^	18.65 (3.41)^a^	19.41 (3.35)a	0.873
	Internal fixation	4.49 (0.47)^ab^	5.23 (0.20)*^ab^	5.08 (0.17)*^ab^	0.002

ROM, range of motion.

Data are presented by mean and standard deviation.

*Indicates a significant difference compared to the expandable device group.

†Indicates a significant difference compared to the Z-Plate group.

^a^Indicates a significant difference compared to the normal state within a group (all, p <0.001).

^b^Indicates a significant difference compared to the injured state within group (all, p <0.001).

After internal fixation, the mean ROM after internal fixation of all 3 systems was similar in right bending (5.01, 5.05, and 5.04; p = 0.887), which is also comparable to Kaneda system in left bending (5.04). After internal fixation, the expandable device and Z-plate system exhibited higher left bending motion than the Kaneda (system 5.50° and 5.37° vs. 5.04, p =0.001 and 0.008, respectively). Furthermore, after internal fixation, the Z-plate and Kaneda groups had significantly higher left axial and right axial rotation ROM as compared to expandable device (left axial rotation: 5.23° and 5.02° vs. 4.53°, p =0.002 and 0.016, respectively; right axial rotation: 5.23° and 5.08° vs. 4.49°, p =0.001 and 0.005, respectively).

### Torque and pullout strength

The Kolmogorov-Smirnov test for normality indicated that all data were normally distributed (Additional file 1: Table S2). There was no significant difference of maximum insertion torque between the common screw group and the expandable device unexpanded group. The maximum insertion torque of the expandable device was significantly greater than of the common screw (5.10 vs. 3.75 Ns, respectively, p =0.005) (Table 2). The differences of maximum pullout force among the 3 groups reached statistical significance (p <0.001) (Table 2). The maximum pullout force of the expandable device unexpanded was significantly greater than of the common screw (2333.49 N vs. 1827.38 N, respectively, p =0.006). The maximum pullout force of the expandable device expanded was significantly greater than of the common screw group and the expandable device unexpanded (3035.48 N vs. 1827.38 N and 2333.49 N, respectively, p <0.001).

**Table 2:** **Torque and pullout strength**

	Common screw (n = 6)	Expandable device unexpanded (n = 6)	Expandable device expanded (n = 6)	p
Maximum insertion torque (Ns)	3.75 (0.69)	4.50 (0.56)	5.10 (0.83)*	.016
Maximum pullout strength (N)	1,827.38 (260.38)	2,333.49 (310.14)*	3,035.48 (252.04)*†	<0.001

Data are presented by mean and standard deviation.

*Indicates a significant difference compared to common screw group.

†Indicates a significant difference compared to the anterior fixation screw unexpanded group.

## Discussion

This study examined the biomechanical properties of a newly designed built-in expandable anterior spinal fixation system. The results showed that the 3D ROM afforded by the new device was similar to that of the Z-Plate and Kaneda systems, though the Z-plate and Kaneda groups had significantly higher left axial and right axial rotation ROM as compared to expandable device. The maximum torque of the expandable device was greater than that of a common screw, as was the maximum pullout force of the device in both the unexpanded or expanded state.

With the built-in expandable anterior thoracolumbar fixation system the vertebral screw and the rod are completely implanted within the vertebra. This avoids disturbing blood vessels, nerves, and soft tissue around the vertebra, thus overcoming a shortcoming of the screw-rod system. The connecting rod is closer to the center of mechanical transmission, and its torque is relatively short compared to traditional internal fixation devices, which prevents easy screw breakage. In addition, implantation is not affected by the shape of the vertebra, which overcomes a shortcoming of the screw-plate system. Because the system is completely built-in, it can be implanted via the lateral, anterolateral, and anterior approaches. Expansion of the distal end of the fixator increases both the holding force and the anti-rotation capacity of the vertebral screw, overcoming the poor anti-rotation capacity of the single screw-rod system. The aperture on the leaflet in the distal end of the vertebral screw, and the gap between leaflets, connect bone fragments within the vertebral screw and bone outside of the screw, thus promoting bone ingrowth to achieve true permanent fixation, i.e., the system is designed such that the implant and vertebral body become fused together, and thus the implant cannot be removed. Lastly, the vertebral screw is designed to have a long tail, which can be used for distraction and compression of the intervertebral disc space, and can be broken off after surgery.

Fresh adult pig spines are similar to the human spine, they are easily obtained, and specimens of the same weight, age, and size are simple to identify. For these reasons the porcine spine is an ideal animal specimen for testing the biomechanical properties of internal fixation devices in the thoracolumbar spine [16]. The characteristics of spine motion indicate that the load of the specimen is the moment applied to the cephalic and caudal ends. Because of the complexity and individual differences of *in vivo* spine loading, the moment is closely related to the amount of regular exercise an individual receives. In addition, the moments on different segments of a spine specimen are different. The loaded torsional moment can at least guarantee normal ROM of a spine specimen.

Though the ROM of the whole spine is large, the amplitude of the motion of each segment is relatively small. The true load-deformation curve of a functional spinal unit is 2-phase and non-linear [15]. At the initial portion of the curve, the deformation is relatively small and the corresponding gradient is also relatively small. As the load increases, the deformation resistance increases. The first stage of motion, the initial portion of the curve with a small gradient, is termed the NZ. When the load increases and exceeds the limitations of the NZ, the resistance to deformation increases significantly. This is the second stage of the curve, and is termed the EZ. The sum of the NZ and EZ represent the total physiological activity (ROM) of a functional spinal unit [15]. However, evaluation of a multi-segmental spinal unit (MSU) is more valuable when examining the biomechanical effect of different instrumentation systems [13, 15].

Internal fixation of the spine mainly aims to provide sufficient stability before rigid fusion of the spine occurs [1, 2]. High immediate stability after internal fixation increases the fusion rate of interbody bone grafting [17], and reduces the rate of internal fixation failure. All spinal fixation systems provide biomechanical stability; however, stiffness of the spine after instrumentation has been shown to be related to the design of instrumentation system, rather than if it is a plate or rod style system [18, 19].

We found that the maximum torque and pullout force of the expandable device were greater than that of a common fixation screw. Both anterior and posterior internal fixation screws are affected by axial pullout force, flexion force, and rotation force. Screw loosening and pullout are the result of the combined effects of these 3 forces. Most biomechanical experiments use axial pullout force as an indicator of screw holding strength [20]. Screw pullout force is related to screw shape, diameter, and depth of insertion, and bone density, and the most important factors are screw diameter, length, and bone density [20]. All screws used in the current study were 30 mm in length, which eliminated the effect of screw length on the results. The maximum axial pullout force (F-max) of the screw depends on the shear stress between the screw and bone; namely, when the contact area between the screw and the bone is large, the shear stress is large. Liu et al. [21] consider that axial pullout of a screw may induce a torque ‘spinning out’ the screw because there is a certain inclination in the screw thread, and this may accelerate screw loosening. Therefore, when only the axial pullout force is examined there is a limitation in the evaluation of anterior screw stabilityRotational torque depends on the friction at the interface between the screw and the bone, and is represented by f = μ × N, where f is the force of friction, μ is the friction coefficient of the screw-bone interface, and N is the positive pressure of the screw-bone interface. The rotational torque of a common screw depends on the force at the screw-bone interface, and its size is relatively limited.

In general, pullout force increases with enlargement of the screw diameter. In a cadaveric study, Willett et al. [22] reported that the mean pull-out force for a 6-mm screw was 597 N, significantly greater than the mean force of 405 N for a 5 mm screw. Large diameter screws compress the cancellous and trabecular bone during screw insertion to form a dense bone layer, and thus the screw thread is embedded in dense bone thereby increasing the pullout force of the screw [23]. Study has shown no significant difference in the maximum axial pullout force of screws less than 1 mm in diameter, but there is a significant difference when the screw diameter is more than 1 mm [24]. Krag et al. [25] reported that screwing-in torque increased significantly when the diameter of a screw increased from 6 mm to 8 mm.

The diameter of the unexpanded screw in the current study was 6.5 mm, which is larger than that of common screws, and the diameter of the screw tip becomes larger after expansion. The F-max of the common screw was 1,826.67 ± 260.25 N, that of the unexpanded anterior fixation screw was 2,333.49 ± 310.14 N, and that of the expanded anterior fixation screw was 3,035.48 ± 252 N, which was 30.09% more than the pullout force of the unexpanded screw and 65.14% more than that of the common anterior fixation screw. By inserting an inner core into the expandable anterior thoracolumbar fixation screw, the screw tip expands. This increases the screw diameter to increase the holding force, and pushes the leaflet in the distal end of the screw to create a “claw” shape that increases the contact area with the bone, also increasing the holding force.

A greater pullout force is especially important in patients with osteoporosis. Study has shown a positive correlation between bone mineral density (BMD) and F-max; namely, the greater BMD, the greater the fixation strength [26]. Li et al. [27] defined a BMD below 0.9 g/cm^2^ as osteoporosis, and found that the F-max and bending moment of screws were 1,062.8 ± 72.2 N and 2.6 N^.^m, respectively, in bone with a BMD above the cutoff, and 232 ± 92.4 N and 0.49 N^.^m in osteoporotic bone. Okuyama et al. [26] reported that the F-max of a screw decreases 60 N when the BMD decreases 0.01 g/cm^2^ in osteoporotic vertebrae. Similarly, Yamagata et al. [28] reported that the F-max of a screw decreases l0 kPa when the BMD decreases 100 mg/cm^2^. Cook et al. [29] applied expandable screws to human spine specimens and found that there was positive correlation between BMD and F-max. In the current study, we used a randomized design to help exclude the impact of bone density on the results.

There are limitations of this study that should be considered. The number of specimens tested was small, and while fresh adult pig specimens closely resemble the human spine, the number of thoracic vertebra is not the same as in the human spine (the pigspine has 15 thoracic vertebra). Because this study used pig spine specimens, and the absolute values for torque and pullout force will be different than if testing were done *in vivo*. Because the diameter of the screws used in the MAC system is large and can damage the vertebral body, we did not include MAC system in the study. In an adult thoracolumbar spine screws 6 mm or 6.5 mm are commonly used. In China, the diameter of most screws used with the thoracolumbar anterior single screw-rod system are 5.5 mm, which is why 5.5 mm screws were used in this study. The difference in screw diameter will have affected the pullout strength results. In this study the pull- out strength of a single screw was compared among the 3 systems. The results may be different clinically as in the Kaneda and Z-plate 2 screws are used in each vertebra which may increase the overall pullout force. We did not perform fatigue testing or 3-point bending testing of the new system. Lastly, a finite element analysis should be carried out to study the load-carrying capacity of the screw, and provide a theoretical basis for further improvement of the design.

## Conclusions

The built-in anterior fixation system tested provides better axial rotational stability as compared to other 2 systems tested, and greater maximum torque and pullout strength than a common fixation screw. The system can be installed with less manipulation of surrounding tissue than rod or plate systems.

## Electronic supplementary material

Additional file 1: Table S1: Normality test for spinal range of motion. Table S2 Normality test for torque and pullout strength. (DOC 60 KB)

Below are the links to the authors’ original submitted files for images.

Authors’ original file for figure 1

Authors’ original file for figure 2

Authors’ original file for figure 3

Authors’ original file for figure 4

Authors’ original file for figure 5

Authors’ original file for figure 6

## Abbreviations

ANOVA: Analysis of variance
AO screw: Algemeinshaft fur osteosynthesefragen screw
BMD: Bone mineral density
EZ: Elastic zone
LSD: Least-significant difference
MSU: Multi-segmental spinal unit
NZ: Neutral zone
ROM: Range of motion
PLC: Posterior ligament complex.
