# Supplementary material for: Biomechanical testing of a unique built-in expandable anterior spinal internal fixation system
Source: BMC Musculoskelet Disord. 2014 Dec 11;15:424. doi: 10.1186/1471-2474-15-424 (PMC4295300; doi:10.1186/1471-2474-15-424)
Supplement: Supplementary file 1 — Additional file 1: Table S1: Normality test for spinal range of motion. Table S2 Normality test for torque and pullout strength. (DOC 60 KB) [file 12891_2014_2384_MOESM1_ESM.doc]

Supplemental Table 1. Normality test for spinal range of motion

|  | |  | Kolmogorov-Smirnov test for normality | | |
| --- | --- | --- | --- | --- | --- |
|  |  |  | Statistic | df | p |
| Extension | Normal |  | 0.104 | 18 | 0.200 |
|  | Injury |  | 0.176 | 18 | 0.148 |
|  | Internal fixation |  | 0.124 | 18 | 0.200 |
| Flexion | Normal |  | 0.124 | 18 | 0.200 |
|  | Injury |  | 0.172 | 18 | 0.171 |
|  | Internal fixation |  | 0.139 | 18 | 0.200 |
| Left axial | Normal |  | 0.126 | 18 | 0.200 |
|  | Injury |  | 0.109 | 18 | 0.200 |
|  | Internal fixation |  | 0.110 | 18 | 0.200 |
| Left bending | Normal |  | 0.156 | 18 | 0.200 |
|  | Injury |  | 0.148 | 18 | 0.200 |
|  | Internal fixation |  | 0.095 | 18 | 0.200 |
| Right axial | Normal |  | 0.130 | 18 | 0.200 |
|  | Injury |  | 0.111 | 18 | 0.200 |
|  | Internal fixation |  | 0.190 | 18 | 0.084 |
| Right bending | Normal |  | 0.126 | 18 | 0.200 |
|  | Injury |  | 0.111 | 18 | 0.200 |
|  | Internal fixation |  | 0.146 | 18 | 0.200 |

Supplemental Table 2. Normality test for torque and pullout strength

|  |  | Kolmogorov-Smirnov test for normality | | |
| --- | --- | --- | --- | --- |
|  |  | Statistic | df | p |
| Maximum insertion torque |  | 0.111 | 18 | 0.200 |
| Maximum pullout strength |  | 0.128 | 18 | 0.200 |
