# Supplementary figures and images for: Biomechanical testing of a unique built-in expandable anterior spinal internal fixation system
Source: BMC Musculoskelet Disord. 2014 Dec 11;15:424. doi: 10.1186/1471-2474-15-424 (PMC4295300; doi:10.1186/1471-2474-15-424)

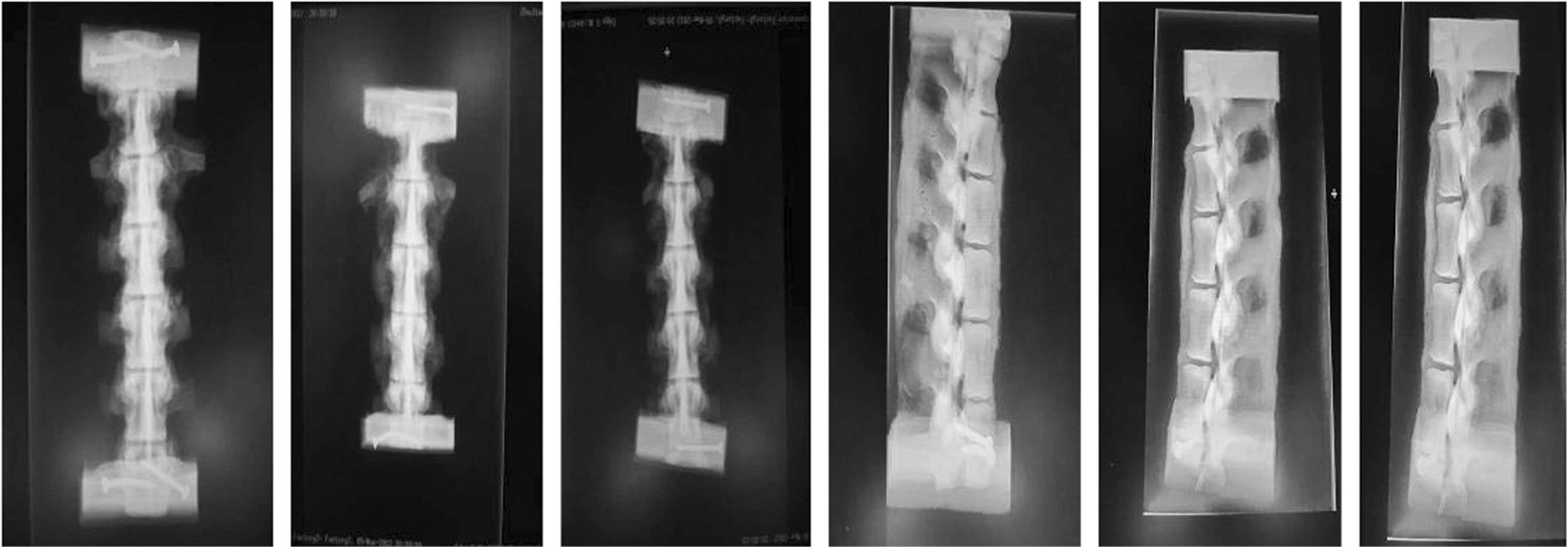

Supplement: Supplementary file 2 — Authors’ original file for figure 1 [file 12891_2014_2384_MOESM2_ESM.tif]

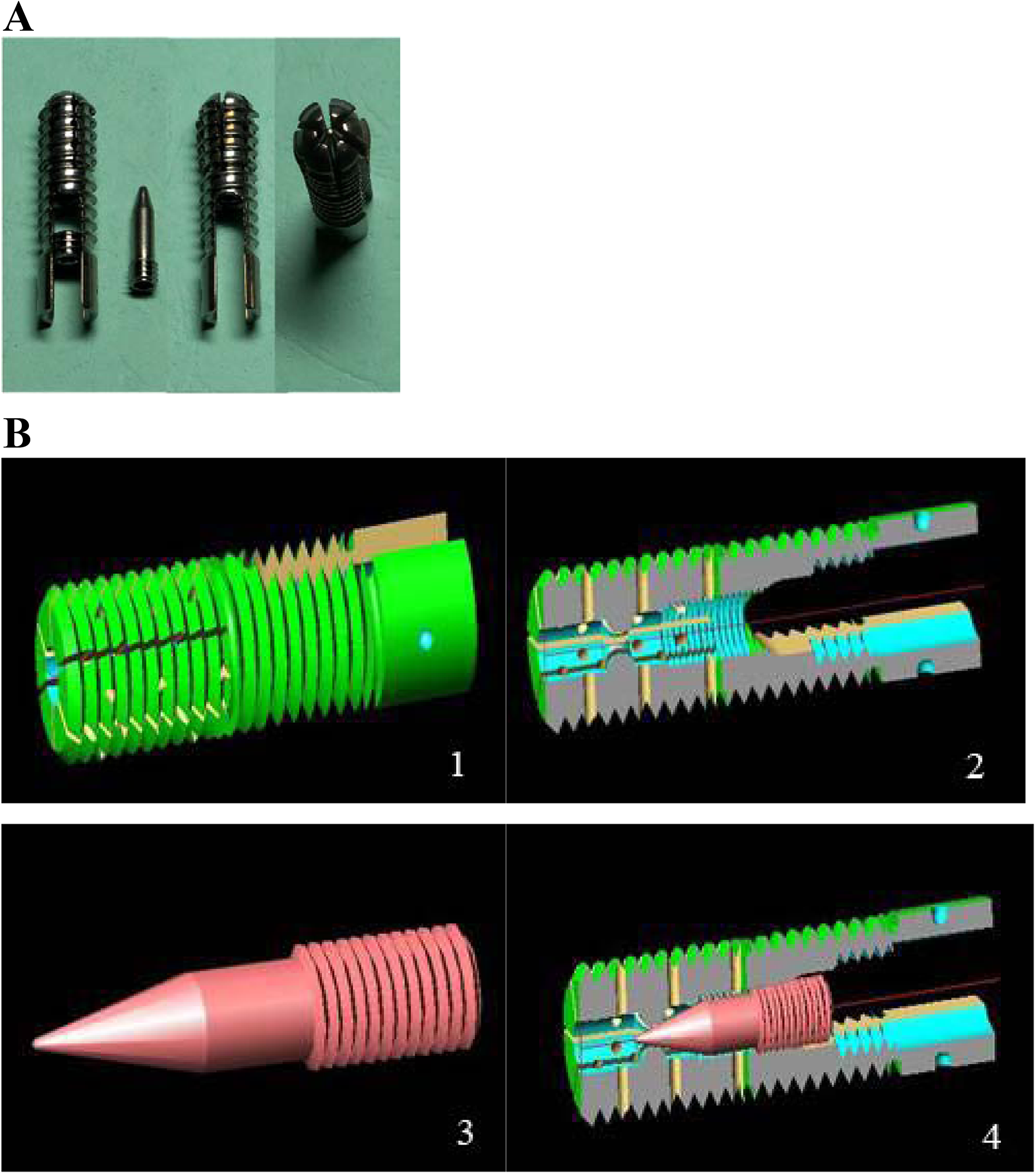

Supplement: Supplementary file 3 — Authors’ original file for figure 2 [file 12891_2014_2384_MOESM3_ESM.tif]

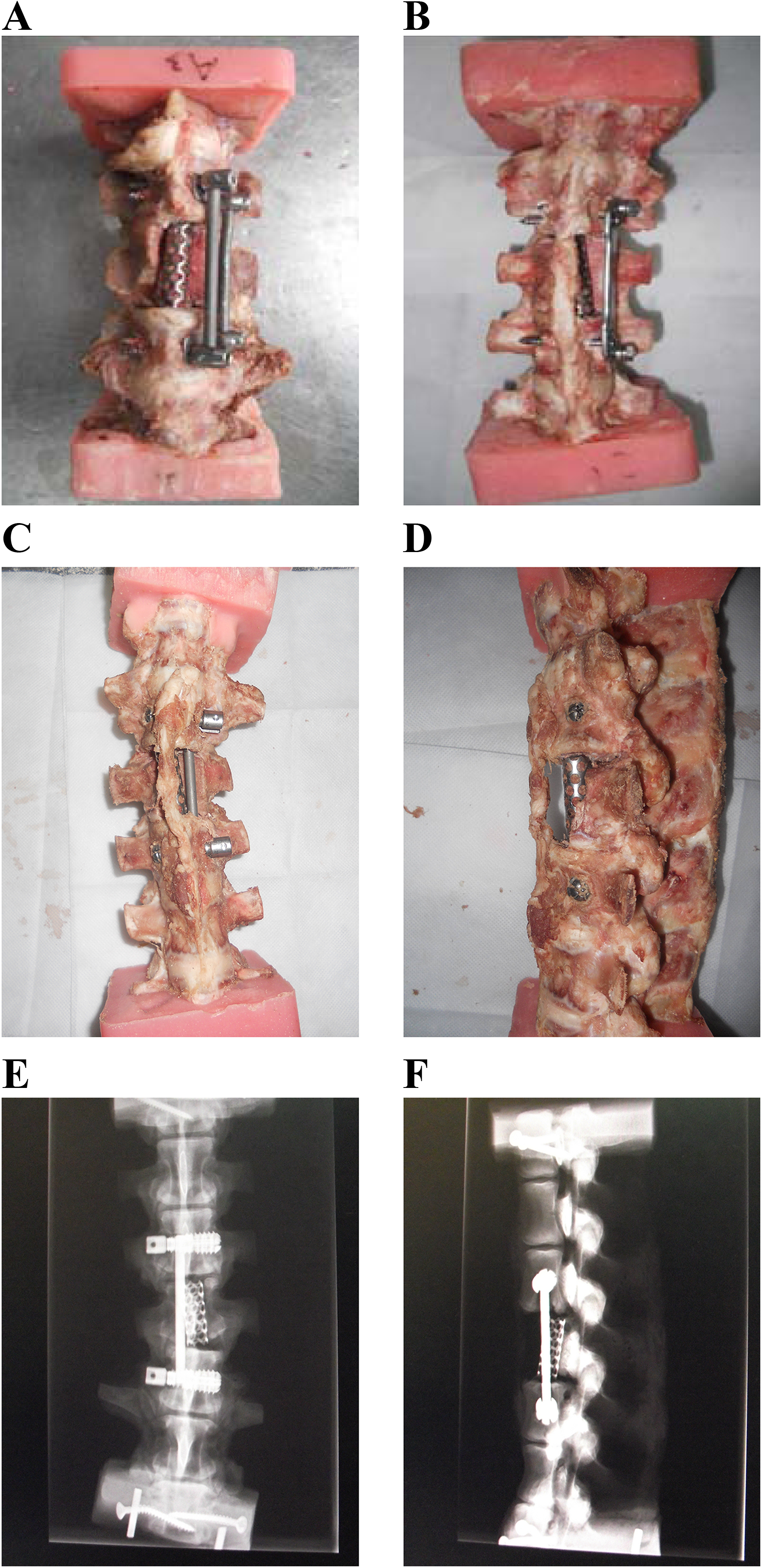

Supplement: Supplementary file 4 — Authors’ original file for figure 3 [file 12891_2014_2384_MOESM4_ESM.tif]

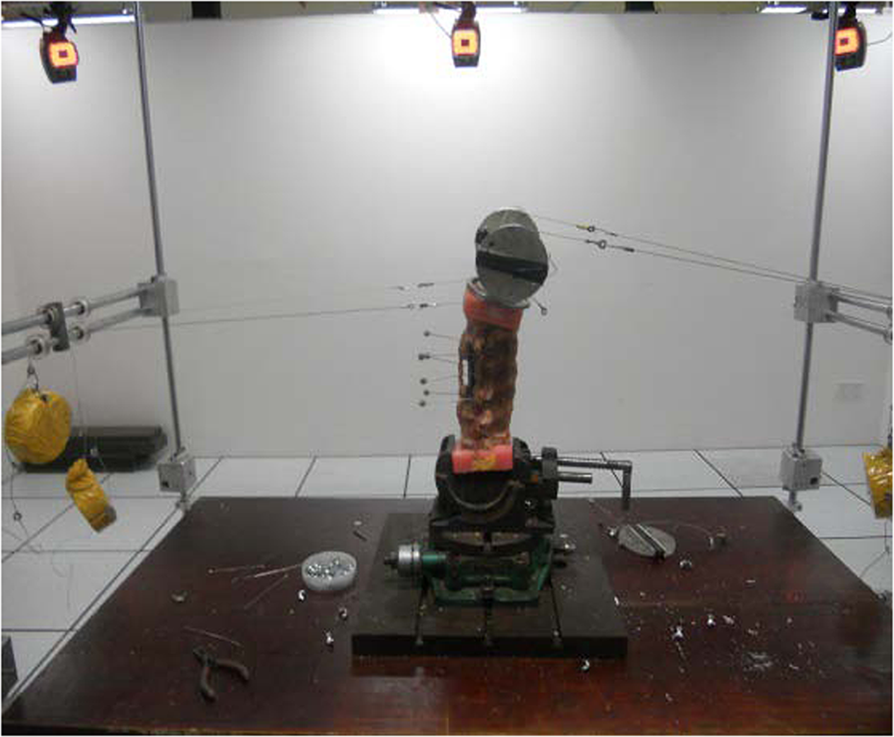

Supplement: Supplementary file 5 — Authors’ original file for figure 4 [file 12891_2014_2384_MOESM5_ESM.tif]

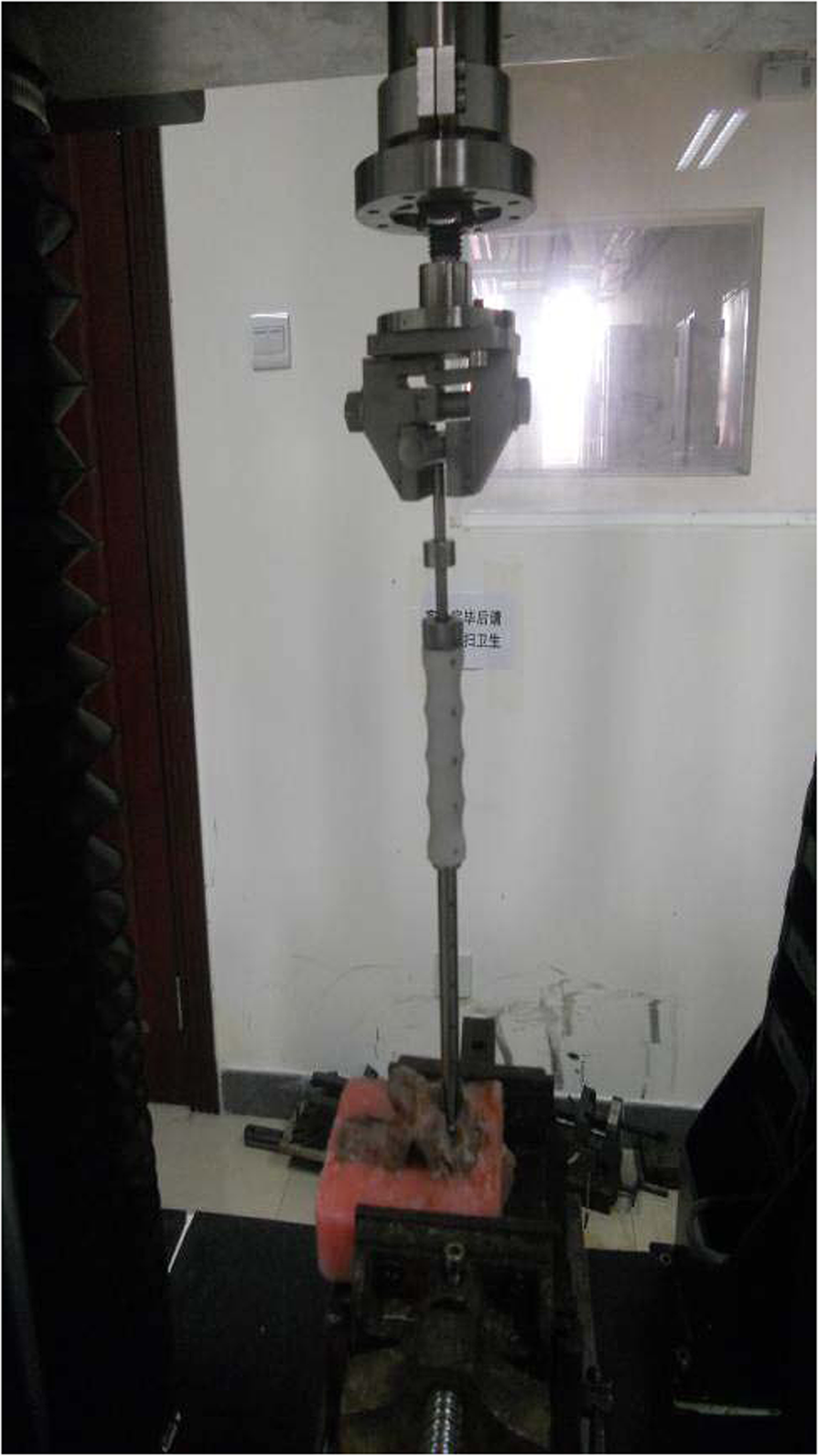

Supplement: Supplementary file 6 — Authors’ original file for figure 5 [file 12891_2014_2384_MOESM6_ESM.tif]

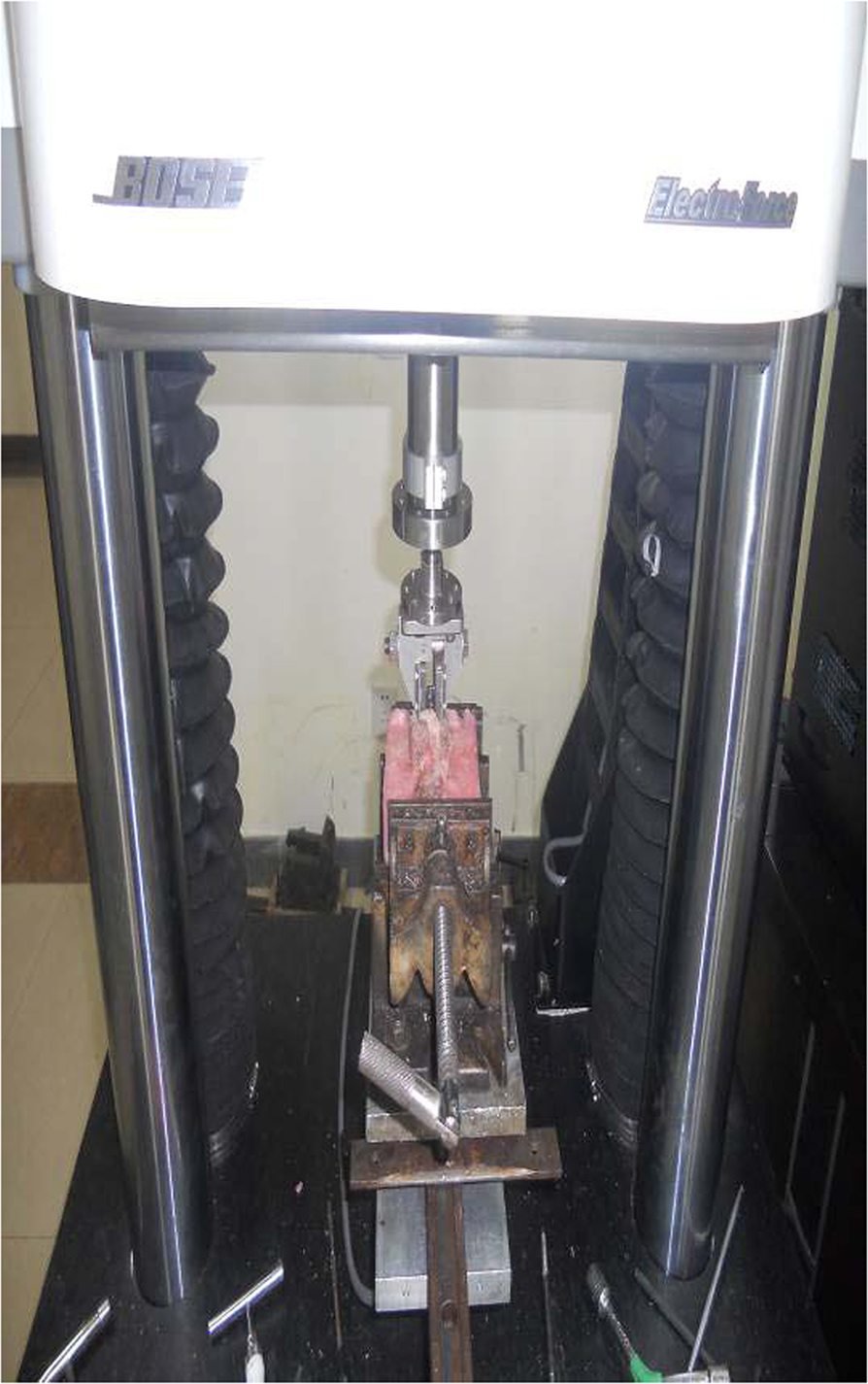

Supplement: Supplementary file 7 — Authors’ original file for figure 6 [file 12891_2014_2384_MOESM7_ESM.tif]
